# Radiofrequency and Electrical Muscle Stimulation: A Synergistic Treatment That Achieves Lipolysis and Circumferential Waist Reduction in Noninvasive Body Contouring

**DOI:** 10.1093/asjof/ojae042

**Published:** 2024-05-28

**Authors:** Neil M Vranis, Ashkan Ghavami, Rodney J Rohrich, Spero Theodorou

## Abstract

**Background:**

Surgeons and providers in aesthetic medicine seek noninvasive devices that can be utilized for safe, efficient, and effective body contouring. Patient demand has propelled the development of novel devices that can simultaneously improve skin laxity, adipolysis along with stimulation of muscle hypertrophy.

**Objectives:**

To determine the efficacy of body contouring after 3 treatments using the noninvasive Transform (InMode, Lake Forest, CA) device.

**Methods:**

A prospective, multicenter study was performed. Outcomes evaluated include: standardized caliper and ultrasound measurements of abdominal skin/soft-tissue thickness, waist circumference, histologic evaluation, patient comfort, and satisfaction assessments.

**Results:**

Forty-four patients were successfully enrolled in the study and completed the series of 3 treatments which involved combined electrical muscle stimulation (EMS) and noninvasive bipolar radiofrequency (RF). Abdominal ultrasound measurements reveal a decrease in soft-tissue thickness (average 3.1 mm; *P* = .001), there was a significant decrease in caliper measurements of periumbilical skin thickness (*P* < .003), and the average reduction of abdominal circumference was 1.9 cm (*P* < .0001) 3 months after the treatment series. Histology confirmed subcutaneous adipolysis without damaging the dermal layer. Patients reported a high degree of satisfaction with the overall result (*P* = .003) and that each of the 3 treatments were progressively more comfortable (*P* < .005).

**Conclusions:**

This study demonstrates that a series of simultaneous noninvasive RF with EMS treatments to the abdomen decreases subcutaneous soft-tissue thickness of the treated area. These comfortable treatments ultimately result in a high degree of patient satisfaction at 3 months.

**Level of Evidence: 4:**

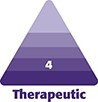

The ideal aesthetic components of the abdomen and flank soft-tissue envelope include taught skin with minimal adiposity to highlight the well-defined underlaying muscle/bone contours.^1^ Noninvasive body contouring modalities traditionally addressed adipose reduction with minimal secondary impact on improving skin laxity or muscular hypertrophy. Numerous technologies have been used to improve lipodystrophy, with the most well-known example, including cryolipolysis (CoolSculpting, Allergan, Dublin, Ireland).^1-4^ Although studies demonstrate modest improvements in adiposity, issues of secondary skin laxity as well as the poorly understood phenomenon of paradoxical adipose hyperplasia that can occur limit its utility.^5^

In the mid-2000s, electrical muscle stimulation (EMS), a technology previously used exclusively in rehabilitation medicine,^6-8^ gained popularity within aesthetics because of the “effortless” development of muscle hypertrophy. Imaging studies confirm sustained improvements in muscle hypertrophy.^9,10^ However, the aesthetic utility of EMS devices falls short because of the residual lipodystrophy in many patients.^6,11^ Naturally, there has been a rise in patient and provider interest in a more comprehensive noninvasive body contouring solution. Radiofrequency (RF) technology in combination with EMS is of particular interest to simultaneously and synergistically address dermal laxity, adipose excess, and muscle tone.^12^

RF has been utilized in many medical fields. The use of electrical current to generate heat through Ohm's law (*V* = *IR*) in soft tissues, preferentially targeting collagen and adipose cells, has been successfully used for aesthetic purposes, such as fat reduction, dermal/subdermal tightening, and even improvements in the appearance of cellulite.^13-17^ The ability to safely and reproducibly deliver RF energy in a bipolar mechanism with precise real-time thermal control has significantly advanced the safety and use of RF over the past years.^13-17^ The capability to volumetrically heat tissues using RF is a favorable combination with EMS for 2 reasons: (1) heat acclimation (ie, preheating muscle) allows for a more efficient contraction and faster recovery and (2) simultaneous intensive muscle workload increases lipolysis efficiency.^18,19^

The purpose of this study was to evaluate clinical and histologic results after a treatment series that used alternating bipolar RF and EMS. This comprehensive study was multicentered and prospective. Previous publications have used preliminary data to demonstrate basic safety of the Transform (InMode, Lake Forest, CA) device.^12,20^ This study was appropriately powered with primary endpoints to objectively measure and assess adipose soft-tissue thickness, changes in waist circumference, and histologic changes of the skin and subcutaneous tissue. Secondary endpoints include subjective patient outcomes, such as overall satisfaction with body contouring and degree of comfort with each treatment session.

## METHODS

This IRB-approved study (Sterling Institutional Review Board; Clinical Trial Protocol Number: NCT05398159) included 5 sites (TN, TX, PA, NC), with all patients independently presenting to each practice desiring noninvasive body contouring. They voluntarily consented to participation in this study. Data for this study were collected from March 2021 to September 2022. A power analysis (alpha <0.05, power = 0.95) dictated that at least 43 (*n* = 43) patients needed to be included to detect a moderate effect.

The study included patients without any comorbidities, interested in noninvasive abdominal body contouring, and who were between the ages of 18 to 70 years with a BMI ≤30. They were excluded if they were pregnant, breastfeeding, had implantable electrical medical devices, history of cancer, or coagulopathy. All patients were required to return to clinic for the 3-month follow-up appointment. Patients with weight fluctuations >3% of their baseline bodyweight were excluded from the study. To maintain consistency across treatments, a narrow window of settings was applied. Providers were instructed to set the device to with 41°C gradually increasing to 43°C according to patient comfort. The EMS power level was set according to the patient's tolerance, starting at intensity Level 5. Increase gradually to visible muscle contraction.

The efficacy of the Transform (InMode) treatment was assessed across numerous outcomes. Soft-tissue thickness was assessed using a caliper to measure the thickness of soft-tissue “pinch” as well as an ultrasound measurement (from dermis to rectus fascia) while soft tissue was in its native position. The location of these measurements was also recorded for each patient to minimize sampling error. Triangulation of “fixed points” (ie, umbilicus, moles, scars, tattoos, etc) was utilized. Torso circumference was recorded at the level of the widest diameter with a second measurement 2 inches above and a third measurement 2 inches below (both are parallel to the initial measurement). Subjective patient comfort ratings during the treatment as well as satisfaction with the overall results were collected and analyzed. One-sample *t*-test, paired samples *t*-test, or within-patients repeated measures analysis of variance was conducted. The analysis was carried out using R-4.3.1 (R Foundation for Statistical Computing, Vienna, Austria).

Waist circumference measurements were obtained at the widest point between xyphoid and pubis, and additional measurements were taken 2 inches above and below this point. All subsequent measurements taken for each patient were taken at the same distance from the floor to ensure uniformity. Caliper measurements were standardized by taking them from each side of the umbilicus. For the ultrasound measurements, 2 measurements were taken within the treatment area and recoded. The location of each probe was accurately triangulated according to fixed landmarks for each patient (ie, moles, scars, umbilicus, etc). Patient satisfaction and comfort scales were recorded in whole numbers from +2 to −2 with 0 being neutral (Table 1).

**Table 1.** Rating scale to assess patient comfort for each treatment session and overall satisfaction with results at three months after completion of the treatment series.

Comfort Rate	Score
Very Comfortable	+2
Comfortable	+1
Indifferent	0
Uncomfortable	-1
Pain	-2

Satisfaction Rate	Score
Very Satisfied	+2
Satisfied	+1
Indifferent	0
Disappointed	-1
Very Disappointed	-2

Biopsies from the treated areas were collected from the patients who consented to biopsy at baseline and at 3-month follow-up and stained for hematoxylin and eosin, elastin, and Masson's trichrome to assess for changes. Histological analysis was performed to assess changes in the subcutaneous tissue and the safety of the treatment in regards to dermal damage.

## RESULTS

Forty-four (*n* = 44) patients successfully completed the treatment series involving 3 noninvasive sessions of combined bipolar RF and EMS modalities (Videos 1 and 2). The enrolled cohort consisted of 39 females and 5 males. Average age was 47.6 years (range, 24-69 years; SD 12.9). The average height and weight were 5′6″ and 150 lbs (SD 4.0, 21.5), respectively, with an average BMI of 24.2 (range, 18.7-30; SD 2.8). In terms of ethnic origin, the majority of patients were Caucasian (*n* = 33, 75%), followed by Hispanic (*n* = 4, 9.1%), African-American (*n* = 4, 9.1%), Asian (*n* = 2, 4.5%), and Middle-Eastern (*n* = 1, 2.3%).

### Skin and Adipose Tissue Thickness (Ultrasound Measurement)

**Figure 1.**
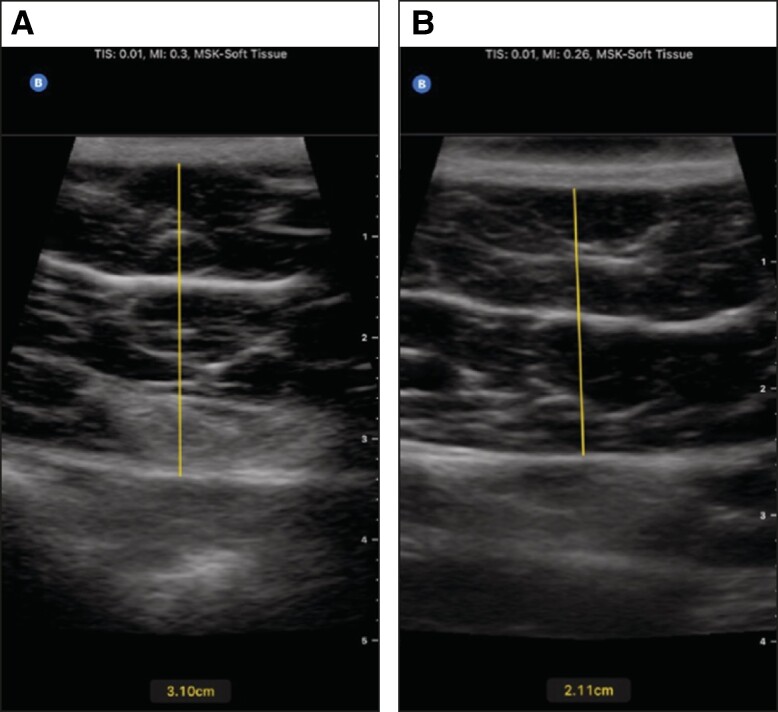
Ultrasound fat thickness measurements at baseline (A) and 3-month after (B) completion of the treatment series. The yellow line depicts the measured adipose thickness with the calculated measurement displayed in yellow font on each image.

Three months after treatment, the reduction in soft-tissue/adipose thickness was reduced by an average of 3.1 mm (Figure 1). The mean ultrasound measurements demonstrated a significant reduction at 3 months (mean = 21.4 mm) compared with baseline (mean = 24.5 mm; *P* < .0001; Table 2).

**Table 2.** Ultrasound measured thickness (in mm) of abdominal skin and subcutaneous adiposity superficial to abdominal wall fascia measured before and 3-months after the completion of the treatment series.

Outcome (Ultrasound)	Pre-Treatment Mean (SD)	3-Month Mean (SD)	Mean Diff (SD)	t-value	p-value
1^st^ Measurement	24.07 (7.76)	19.84 (6.75)	4.24 (7.35)	3.56	0.001
2^nd^ Measurement	23.98 (7.64)	21.06 (6.02)	2.92 (4.21)	4.22	<0.001
Avg Ultrasound	24.28 (7.60)	20.77 (5.78)	3.51 (5.52)	3.82	0.001

### Skin and Adipose Tissue Thickness (Caliper Measurements)

**Figure 2.**
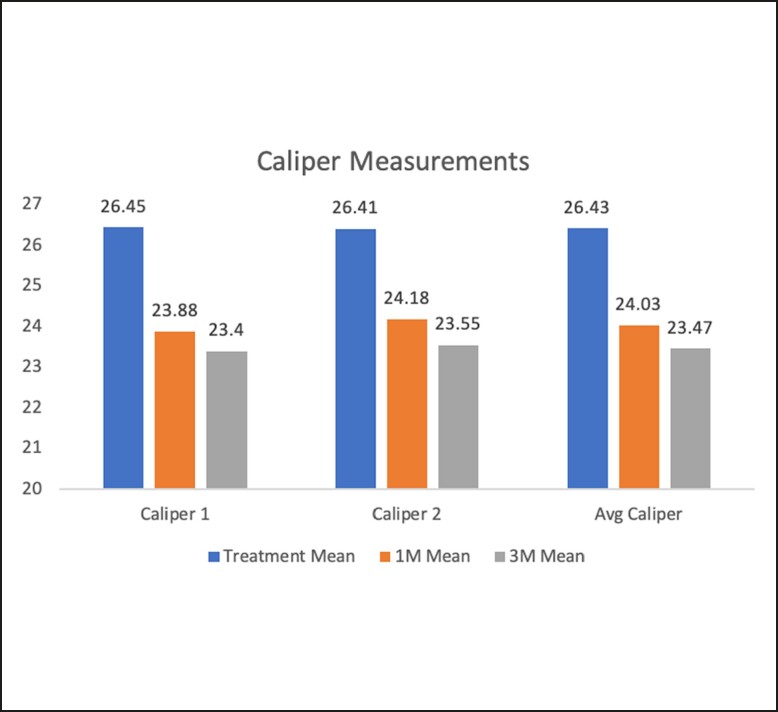
A bar graft depicting the statistically significant improvement in caliper measurements of abdominal skin and soft-tissue thickness 1 month after completion of treatment series and the sustained improvement until the 3 month follow-up visit. Blue bars represent pre-treatment mean values and the orange/grey bars represent the 1 month and 3 month after treatment mean values, respectively.

A second method to assess and confirm a decrease in abdominal skin/soft-tissue thickness was utilizing caliper measurements. The omnibus test was statistically significant for caliper measurements #1, #2, and the average of 1 and 2 measured at 3 different time points: *F*_1_(2, 72) = 7.99, *P* = .001, *F*_2_(2, 72) = 6.42, *P* = .003, *F*_avg_(2, 72) = 7.50, *P* = .001 respectively. A linear, negative correlation was demonstrated from baseline (*m*_1_ = 26.45; *m*_2_ = 26.41, *m*_avg_ = 26.43) to 1-month follow-up (*m*_1_ = 23.88; *m*_2_ = 24.18, *m*_avg_ = 24.03) to 3-month follow-up (*m*_1_ = 23.40; *m*_2_ = 24.18, *m*_avg_ = 23.47). Pairwise comparisons showed that the baseline measurement differed from both subsequent time points. However, there was no difference in measurement at the 1- and 3-month follow-ups. Thus, the improvement of soft-tissue thickness was maintained. Figure 2 displays the mean values across the 3 time points for the 3 caliper outcome variables.

### Waist Circumference Measurements

**Figure 3.**
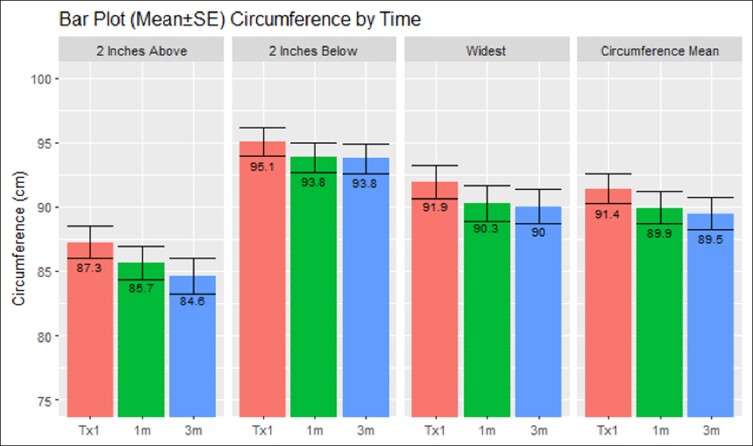
A bar graft depicting that circumference measurements decreased from baseline (red), to 1-month (green), to 3-month (blue) post-treatment for all three measured locations and for their combined average. Pink, green and blue bars represent pre-treatment, 1 month after and 3 months after treatment value, respectively.

The mean circumference measurements demonstrated a significant reduction both at 1 and 3 months (1 month: mean = −1.5 and 3 month: mean = −1.9 cm, *P* = .0017 and *P* < .0001, respectively) compared with baseline. Notably, improvement was apparent during the 1-month follow-up, and this positive trajectory persisted upon reaching the 3-month mark (Figure 3). These findings confirm the presence of a favorable trend in the reduction of circumference over the time following Transform treatment.

### Patient Comfort During Each Treatment

The 5-point scale for patient comfort was objectively evaluated in 2 ways: (1) each of the 3 measurements were compared with a value of 0 (hypothesized to be neutral/indifferent), and (2) comparing comfort scores at each of the 3 time points to one another. Two of the three 1 sample *t*-tests for each of the treatment sessions showed a statistically significant difference (*P*_1_ = .07, *P*_2_ < .001, *P*_3_ < .001). None of the treatments received a score of −2 (ie, were “painful”) for any of the patients (Table 3).

**Table 3.** Patient comfort scores during each of the treatment sessions. (-2 = painful, 2 = very comfortable)

Outcome		Treatment Mean (SD)		t-value	p-value
Subject Comfort Treatment 1			0.30 (1.07)	1.83	0.07
Pain	Uncomfortable	Indifferent	Comfortable	Very Comfortable	
0 (0.0%)	8 (19.5%)	13 (31.7%)	14 (34.1%)	6 (14.6%)	
Subject Comfort Treatment 2			0.71 (0.98)	4.78	<0.001
Pain	Uncomfortable	Indifferent	Comfortable	Very Comfortable	
0 (0.0%)	3 (7.0%)	10 (23.3%)	21 (48.8%)	9 (20.9%)	
Subject Comfort Treatment 3			0.73 (1.02)	4.73	<0.001
Pain	Uncomfortable	Indifferent	Comfortable	Very Comfortable	
0 (0.0%)	6 (14.3%)	7 (23.3%)	21 (50.0%)	8 (19.0%)	

**Figure 4.**
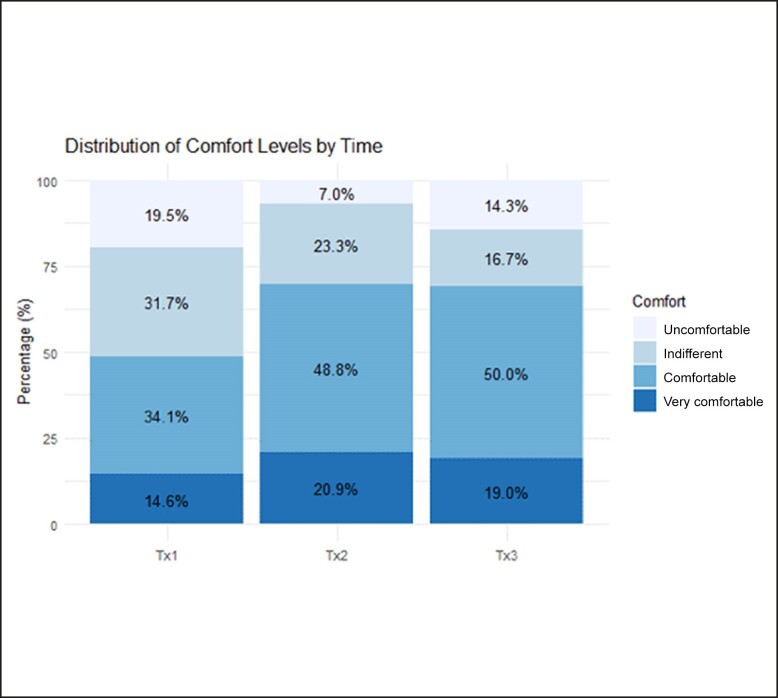
A graphic representation of comfort scores (% of patients) reported for each treatment session. Light grey, teal, light blue and dark blue colors represent patient reported comfort as uncomfortable, indifferent, comfortable and very comfortable, respectively.

The average patient comfort scores improved with each subsequent treatment session despite maintenance of the device settings/level of stimulation. The linear positive correlation was statistically significant, *F*(2, 86) = 5.76, *P* = .004. Pairwise comparisons reveal that each treatment was significantly less painful than the first treatment (Figure 4).

### Patient Satisfaction (3 Months Posttreatment)

**Figure 5.**
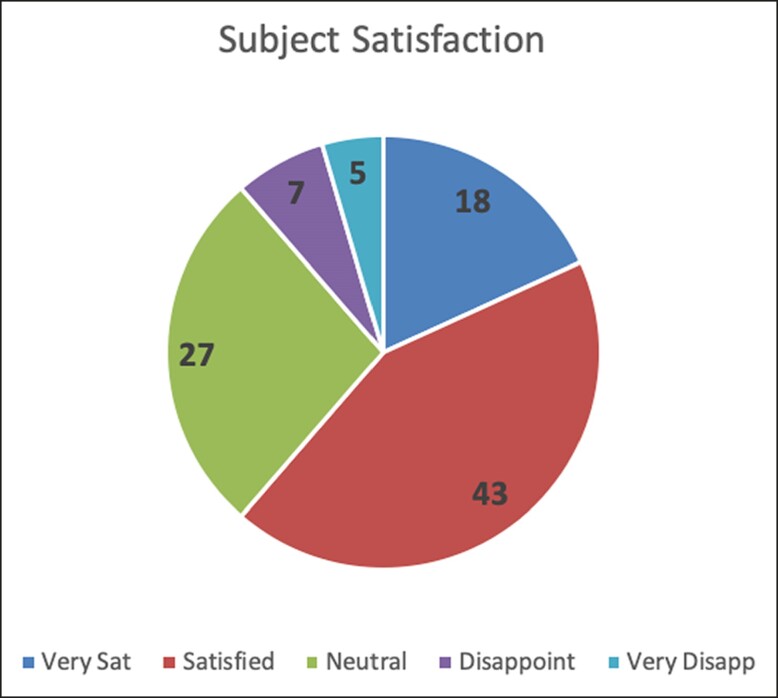
Pie chart depicting the level of patient satisfaction with the clinical outcome after a three-series treatment protocol with the Transform device (InMode, Lake Forest, CA). The dark blue, red, green, purple and light blue sections of the pie-chart represent the proportion of patients who were very satisfied, satisfied, neutral, disappointed and very disappointed with the treatment results, respectively.

**Figure 6.**
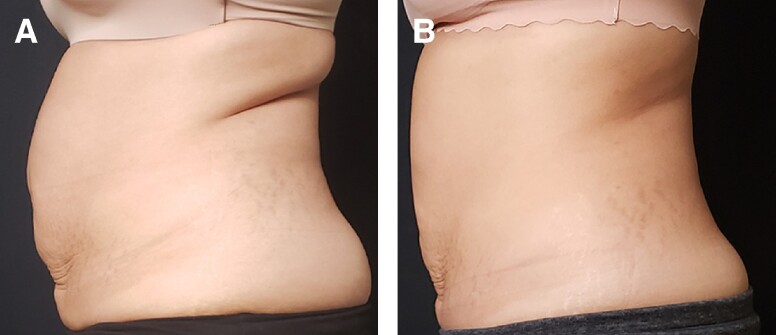
Clinical profile torso photographs of a 36-year old female patient showing the before (A) and six months after (B) treatment series. Improvement in the abdomen and flank is demonstrated in these photographs.

**Figure 7.**
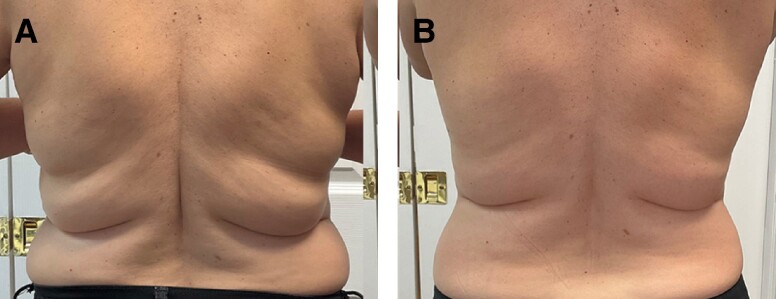
Clinical photographs of a 44-year-old female patient showing the before (A) and six months after (B) treatment series. Adipose reduction and overall contour improvement of the flanks is demonstrated in these photographs.

Patient satisfaction after completion of the treatment series was measured on a 5-point scale that ranged from −2 (very disappointed) to 2 (very satisfied) with a score of 0 representing neutral and baseline for comparison (Table 1). The mean of 0.66 (STD = 1.28) was statistically different from the hypothesized mean, *t*(37) = 3.17, *P* = .003, suggesting that the patients were more satisfied 3 months after the treatment compared with a hypothesized neutral satisfaction level (Figure 5). Clinical photographs highlight the contour changes of the abdomen and flanks after completion of the treatment series (Figures 6, 7).

### Histology

**Figure 8.**
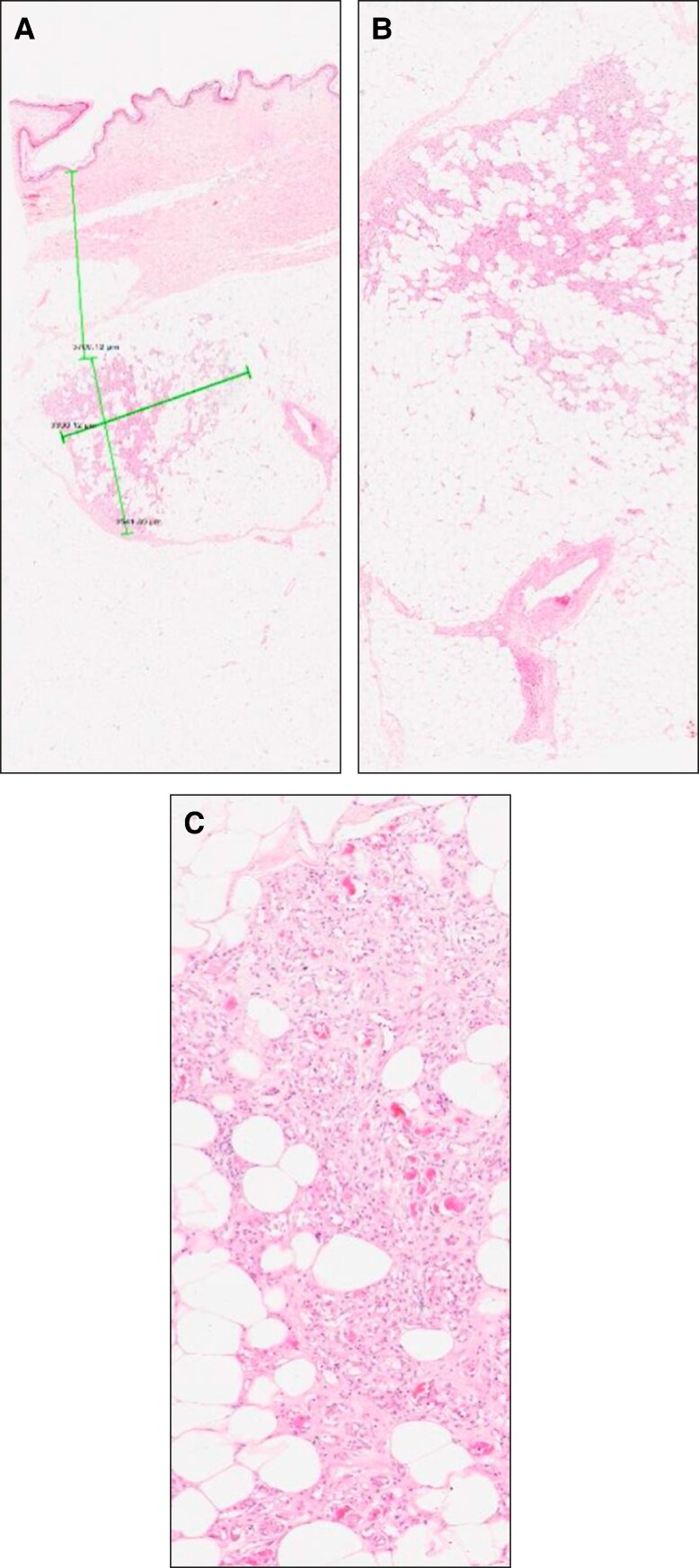
Histology from Patient 1 post-treatment biopsy. An irregularly shaped foci located 4mm below the epidermis (A: 1x magnification, B: 2x magnification). Adipocytes within this focus are separated by inflammatory cells along with fibrous tissue consistent with sub-acute focal panniculitis (C: 10x magnification). The green lines in A represent the area that is further magnified in B and C.

**Figure 9.**
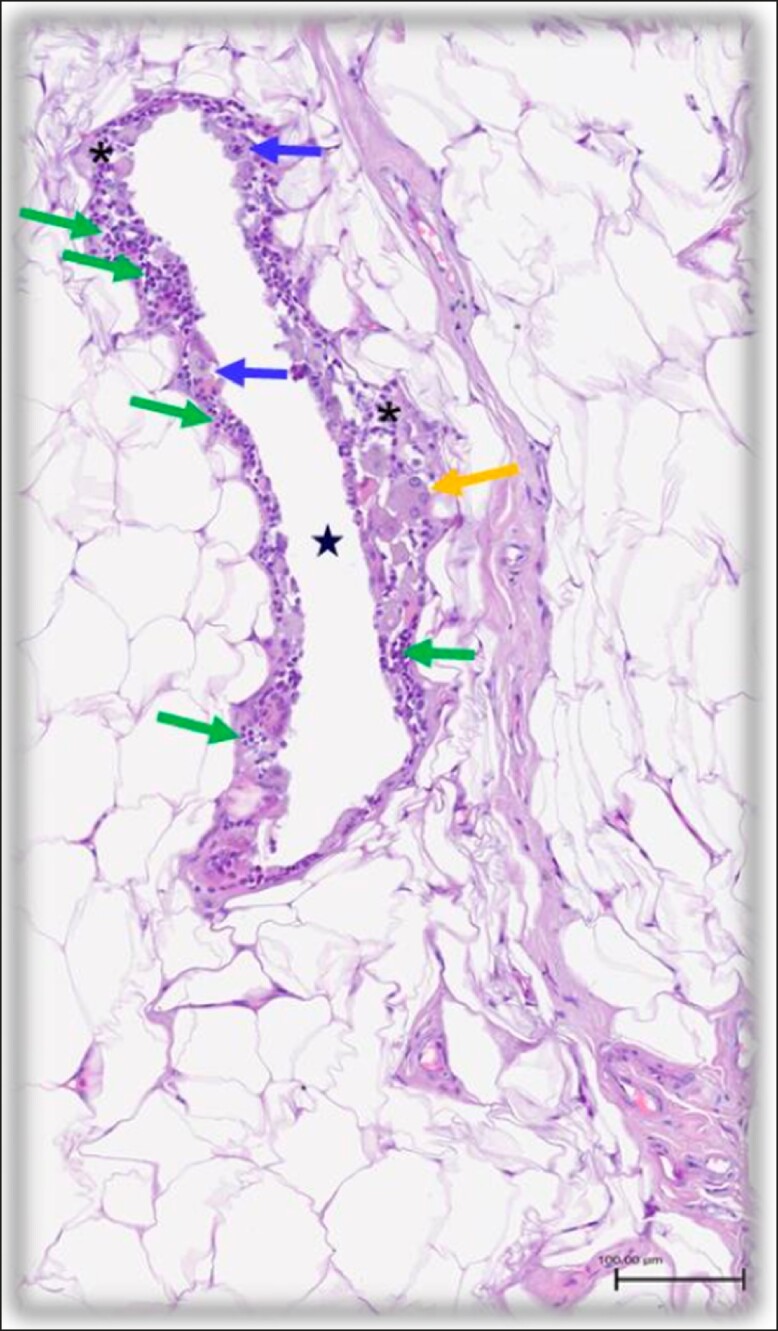
Histology from Patient 2 post-treatment biopsy on high magnification. A clear space of subcutaneous adipocytes volumetric loss (large black star) surrounded by numerous, foamy histiocytes (blue arrows) and lymphocytes (green arrows). A multi-nucleated giant cell with foamy cytoplasm (orange area) is visualized in the adjacent area of inflammation (small black stars).

Abdominal skin/soft-tissue biopsies from within the treatment area were obtained from 2 patients. Biopsies were obtained from each patient before and 3 months after completion of the treatment series. Histologic evaluation of the posttreatment biopsies demonstrated soft-tissue adipocytes surrounded by inflammatory cells (macrophages, neutrophils, and lymphocytes) in addition to fibrous tissue and fibrocytes. Fat necrosis and volumetric loss of subcutaneous adipocytes characterized by clear spaces surrounded by variable amounts of foamy histiocytes and occasional eosinophils and lymphocytes in all treatment sites. Most importantly, the epidermis and dermis were noted to be intact and of appropriate thickness/appearance. Histologically, they appeared to be within normal limits on all evaluated slides (Figures 8, 9).

## DISCUSSION

The results of this study and previous report^12^ demonstrate that combining EMS and bipolar RF modalities using the Transform device (InMode), during a single treatment session is safe and effective for mild noninvasive body contouring. This was the first study to demonstrate that this combination of therapies led to a significant reduction in abdominal circumferential girth. The radiographic and histologic findings aligned with this clinical observation. Noninvasive aesthetic treatments have become increasingly popular over the past 20 years. Both of these modalities have been independently used with different tissue targets and mechanisms of action. Logically, the next frontier involves safely combining synergistic treatments into a single device/treatment in order to optimize treatments for patients and clinicians.

Numerous studies have shown that heating dermal tissue to 42°C triggers a downstream healing cascade that initiates neocollagenesis and elastin formation.^21^ In animal studies, after 10 min of exposure to temperatures of 39°C to 43°C, the amount of collagen increased from an average of 9% before therapy to 25.9% after 3-month follow-up compared with no change in untreated areas.^22,23^ Other studies have similarly shown through electron microscopy that collagen fibrils had a greater diameter after treatment with a single RF session.^24^ Optimizing these forms of noninvasive thermal-inducing treatments is a delicate balance between achieving temperatures that trigger a nonablative wound healing response to remodel collagen as opposed to higher temperatures that may ablate collagen and soft tissue.^13^ RF has been shown to result in skin tightening and reducing adipocytes. Studies investigating the use of RF for subcutaneous fat reduction have ranged from 4.9% to 29.0% in fat reduction.^25-27^ The histologic evaluation in this study affirms these findings, as there was evidence of adipolysis/adipose necrosis at the cellular level which also translated to clinical findings (decreased soft-tissue thickness on ultrasound and skin pinch measurements). It has been shown that with a minimal 15 min exposure time, apoptosis can be initiated with temperatures of 42°C, and exposure time to trigger apoptosis decreases as the temperature rises.^23^ Apoptosis is achieved with temperatures of up to 25°C, whereas higher temperatures may result in immediate cell death or necrosis.^23^

EMS combined with RF provides additional synergistic benefits compared with EMS treatment on its own. It has been well documented that natural, as well as, induced heat acclimatization improves muscle contractility as well as recovery.^28,29^ The EMS functions to produce supraphysiologic muscle contractions through direct stimulation of neuromuscular pathways. This effect not only improves the tone and volume of muscle but also impacts lipolysis because of local energy consumption leading to breakdown to triglycerides stored in fat cells to glycerol and free fatty acids.^23^ As shown in prior studies, the combination of heat and mechanical stimulation of muscle induces significantly higher expression of heat shock proteins as well a myosatellite cells that ultimately lead to muscle hypertrophy and myofiber development.^29,30^ On its own, EMS has been shown to produce an average subcutaneous fat reduction of 19.6% (17.5%-23.3%),^31,32^ an average muscle thickening of 15.1% (14.8%-15.4%),^31,33^ and an average reduction in abdominal separation of 9.95%.^31,32^

Although objective measurements are paramount to affirming the validity of gross clinical observation, patient outcomes are the ultimate endpoint in the fields of aesthetic plastic surgery and body contouring. Less than 10% of patients treated (Figure 9) were disappointed or very disappointed with their results 3 months after the last treatment session. In fact, the majority of the patients were satisfied or very satisfied with their results. There were no adverse effects, highlighting the safety element of this treatment modality. No patient reported the treatment as “painful” in any of the 3 sessions. Given the high degree of patient satisfaction coupled with the safety profile and noninvasive nature of the device, there is a cohort of patients who can benefit from these treatments in the realm of body contouring.

Although this was a multicenter, prospective study, it does have a number of limitations. Despite the adequate sample size (*n* = 44), the use of a control group for comparison—RF alone, EMS alone—may have helped to elucidate the degree of contribution of each technology independently and quantified the synergistic effect of the combination treatment. Optimally, the use of more objective imaging such as computed tomography/MRI would have been preferred to objectively quantify fat vs muscle components of the patient's treatment; however, the unnecessary radiation and diagnostic expense are not warranted, and thus, ultrasound was selected as the preferred imaging modality. As with many body contouring studies, it would have also been optimal to control for possible lifestyle (diet/exercise) confounding variables; however, our strict exclusion criteria of weight maintenance within 3% of baseline was an attempt to mitigate the influence of this confounding variable. Lastly, longer follow-up times will be needed to determine the longevity of treatment efficacy and resolution of subcutaneous inflammation/fat necrosis at a cellular level. Although this study demonstrates a positive response in the subacute time period, additional studies will be needed to assess long-term viability of results or the need for additional maintenance treatment sessions at specific time intervals.

## CONCLUSIONS

Using the Transform device (InMode), we were able to demonstrate a significant reduction in torso circumference and abdominal soft-tissue thickness with high patient satisfaction scores as early as 3 months after completion of the treatment series. Histologic review of the biopsies obtained from the treated areas confirms adipolysis, which aligns with the hypothesized physiologic response after treatment. Most importantly, there were no adverse effects as a result from the simultaneous combination of EMS and RF treatment.
